# A rare case of embolic spondylo-discitis after treatment of aortic valve endocarditis

**DOI:** 10.1186/1757-1626-1-261

**Published:** 2008-10-22

**Authors:** Abhimanyu Beri, Bishav Mohan, Rohit Tandon

**Affiliations:** 1 B301 Clinical Center, 138 Service Road, East Lansing, MI, USA; 2 Dayanand Medical College and Hospital, Tagore Nagar, Ludhiana, India

## Abstract

**Background:**

Infective Endocarditis is associated with a high incidence of embolic events, commonly involving the central nervous system, spleen, kidney, lungs, heart and eyes.

**Case presentation:**

We report a case of infective endocarditis with late embolization to the L_5_/S_1 _region of the spine leading to spondylo-discitis. The disc space infection presented ten days after completion of antibiotic therapy based on blood culture and antibiotic sensitivity.

**Conclusion:**

This is the first reported case of acute infective spondylo-discitis demonstrated on MR imaging following completion of appropriate antibiotic therapy.

## Background

Infective Endocarditis (IE) is associated with a high incidence of embolic events, ranging from 10–49% [1]. Common sites include the central nervous system, spleen, kidney, lungs, heart and the eyes [2]. We report a case of infective endocarditis with embolization to the L_5_/S_1 _region of the spine, despite appropriate intravenous antibiotic therapy.

## Case presentation

**Figure 1:**
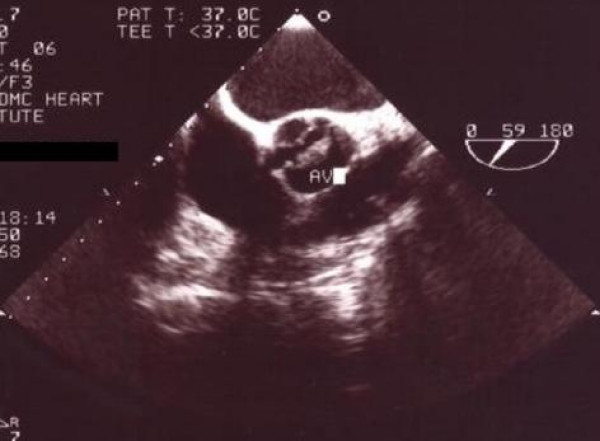
Long axis transthoracic echocardiography of patient showing echogenic mass on ventricular side of aortic valve attached to the right coronary leaflet (vegetation).

**Figure 2:**
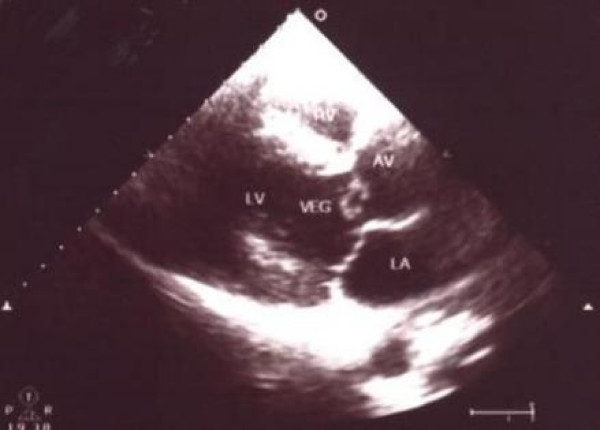
Short axis view on transesophageal echocardiography with valve thickening and an attached echogenic mass (vegetation).

**Figure 3:**
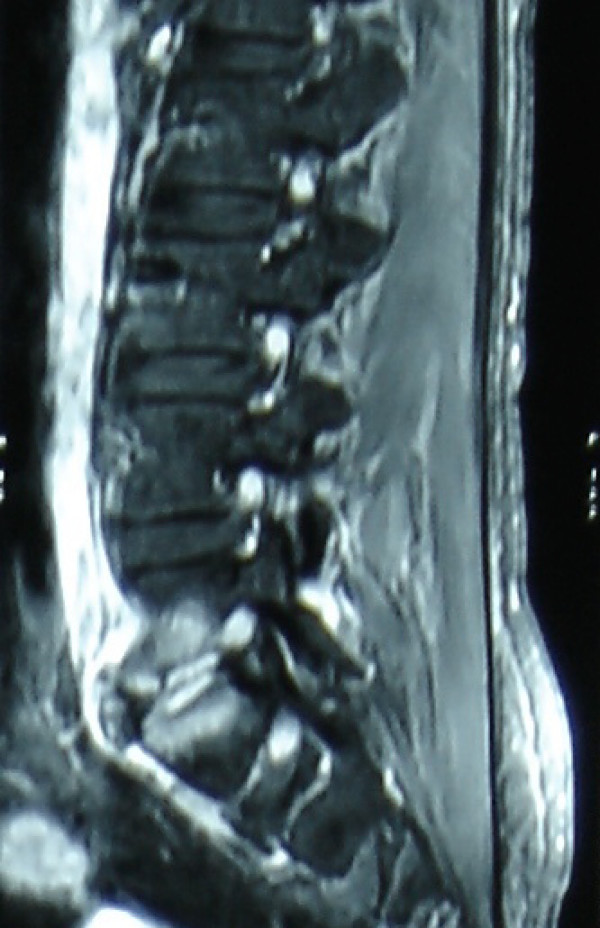
MRI (STIR sequence) showing hyperintense signal from L_5 _and S_1 _vertebrae and the intervertebral disc suggestive of acute infective discitis.

A 38-year-old male presented to out patient department with 10-day history of intermittent fever. The patient had an uneventful inguinal hernia surgery two weeks prior to the onset of fever. Physical examination revealed the presence of a Grade III/VI decrescendo, high-pitched diastolic murmur along the left sternal border. Transthoracic (TTE) and transesophageal echocardiography (TEE) showed the presence of a congenitally bicuspid aortic valve with two 4 mm size vegetations (Figure 1, 2). The left ventricle was mildly dilated with moderate to severe aortic regurgitation. Blood cultures were positive for growth of non-hemolytic Streptococci (NHS). The patient was hospitalized and started on intravenous antibiotics (Vancomycin and Ciprofloxacin) based on the antibiotic sensitivity report. These were continued for four weeks. The patient became afebrile and was discharged. Ten days after discharge, the patient had sudden onset of severe lower back pain while bending to lift a weight. The patient came to the emergency where tenderness at the level of L_5_/S_1 _was detected. No neurological deficits were present. MRI (Magnetic Resonance Imaging) of the spine showed Modic Type II end plate changes with hyperintense signals in the L_5 _and S_1 _vertebrae and in the intervening disc on STIR sequences. Associated marrow edema seen as hypointense signal on T1 weighted images. There were no paravertebral collections. These changes were suggestive of acute infective spondylo-discitis (Figure 3). A TEE at that time showed the formation of an aortic valve abscess. Blood cultures performed were again positive for NHS. Appropriate antibiotic therapy was instituted based on sensitivity report for 10 days following which aortic valve replacement surgery was performed. The native valve was excised and a bioprosthetic valve (21 mm Perimount Edwards) was placed. The abscess cavity was scooped out and debrided. Postoperatively, the patient became afebrile and symptomatically better. On follow-up, the patient reported a subjective improvement in his back pain. A repeat MRI 10 weeks after the previous episode of back pain showed resolution of the hyperintense signal previously present at the L_5_/S_1 _level in the STIR images.

## Discussion

The incidence of IE remains high at 1.7–6.2 per 100 000 person years in the US and Europe [3]. It is associated with high in-hospital mortality ranging from 16–25% [1]. There is a lot of morbidity and mortality due to destructive valvular lesions causing valve regurgitation and heart failure, and a high incidence of embolic events. Predisposing factors include damaged native valves, prosthetic heart valves and intravenous drug use. Most patients can be successfully treated with intravenous antibiotics for 4–6 weeks based on blood cultures and organism sensitivity [4]. Surgery is performed when there is intractable congestive heart failure, recurrent embolization and local abscess formation [2].

Emboli often involve major arterial beds, including central nervous system (65%), spleen (49%), kidneys (22%), lungs (16%), extremities (13%) and coronary arteries (2%) [1]. More than 90% of central nervous system emboli lodge in the distribution of the middle cerebral artery. These emboli are associated with a high mortality rate. Most emboli occur within the first 2 to 4 weeks of diagnosis. The rate of embolic events drops significantly during the first 2 weeks of successful antibiotic therapy, from 13 to < 1.2 embolic events per 1000 patient-days [2]. The type of organism, size and number of vegetations, the number of valves involved, and vegetation characteristics (e.g. lack of calcification) predict embolic complications [2,5].

Dizdar et al reported the case of Group B Streptococcal endocarditis leading to arthritis in the left L_5_/S_1 _facet joint in a patient who presented as lower back pain and bacteremia. On investigation, the site of infection was found to be mitral valve endocarditis [6].

Churchill et al had studied 192 of IE. Five cases out of these who presented with lower back pain were found to have lumbar disc space infection. In all these patients, the symptoms of IE appeared after the back pain [7]. However, this series was performed in the pre-MRI era where this diagnosis was clinical. The diagnosis and treatment of infective endocarditis has undergone a lot of change since then. It is also not confirmed whether the valves or the spine was the primary source of bacteremia.

Ours is a rare case of spondylo-discitis following appropriate treatment of IE. He had IE due to NHS in a congenitally bicuspid aortic valve with small size vegetation at the outset. He had none of the above-mentioned high-risk characteristics that are thought to be predictors of an embolic event and was treated with intravenous antibiotics based on culture sensitivity for four weeks. Despite this, the vegetations persisted and he suffered an embolic episode.

This case shows that IE can present with late embolic episodes, even after the completion of appropriate antibiotic therapy. Lower back pain in patients of IE should not be ignored as this can be due to disc space infection. Our report shows that spondylo-discitis can be a source of bacteremia for endocarditis [7] as well as the result of embolization from infected valves to the spine as in the present case.

## Competing interests

The authors declare that they have no competing interests.

## Authors' contributions

AB and BM were the main contributors in writing the manuscript. RT helped in obtaining and selecting Images. All authors read and approved the final manuscript.

## Consent

Written informed consent was obtained from the patient for publication of this case report and accompanying images. A copy of the written consent is available for review by the Editor-in-Chief of this journal.
